# Effect of two educational interventions on primary school teachers’ knowledge and self-reported practice regarding emergency management of traumatic dental injuries

**DOI:** 10.1186/s12903-019-0823-4

**Published:** 2019-06-27

**Authors:** Samaneh Razeghi, Simin Zahra Mohebbi, Mahdia Gholami, Mahgol Mashayekhi, Bita Maraghehpour, Ebrahim Rahnama

**Affiliations:** 1 Research Center for Caries Prevention, Dentistry Research Institute, Tehran University of Medical Sciences, Tehran, Iran; 2 Department of Community Oral Health, School of Dentistry, Tehran University of Medical Sciences, Tehran, Iran; 3 Arak, Iran; 4 Department of Health Promotion and Community Health Sciences, Texas A&M University Health Science Center, College Station, Texas USA; 5 School of Dentistry, Tehran University of Medical Sciences, Tehran, Iran

**Keywords:** Education, School teachers, Injuries, Dentistry, Knowledge, Practice

## Abstract

**Background:**

The present study evaluated the effect of two educational interventions on the knowledge and self-reported practice of primary school teachers regarding emergency management of traumatic dental injuries (TDIs).

**Methods:**

All primary school teachers (*n* = 664) of Arak, Iran were asked to participate in the study. Based on geographic regions, all participants were enrolled in two groups: educational leaflet and oral presentation. At baseline, teachers in both groups were asked to complete an anonymous self-administrated questionnaire consisting of demographics, eight questions on knowledge, and four paper cases on self-reported practice about TDIs. After collecting the questionnaires, interventions, including an oral presentation and an educational leaflet whose contents were prepared based on the most recent scientific evidence, were implemented. One and six months after the interventions, the questionnaire was completed by the teachers again. Repeated measures ANOVA and a linear regression model were used for statistical analysis.

**Results:**

Two hundred and ninety-two teachers participated in all stages of the study (response rate = 64.5%). In one-month follow-up, in both groups, the mean score of knowledge was significantly higher compared to baseline and six-month scores (*P* < 0.001 and *P* < 0.001, respectively). Moreover, in six-month follow-up, the mean score of knowledge was higher compared to baseline (*P* < 0.001) in both intervention groups. No statistically significant difference existed in the mean score of knowledge in three evaluations between two groups (*P* < 0.05). In one-month follow-up, the mean score of self-reported practice was significantly higher compared to baseline and six-month scores in both interventional groups (*P* < 0.001 and *P* < 0.001, respectively). There was no significant difference in the mean score of self-reported practice between the one-month and six-month follow-up (*P* = 0.53). There was no statistical significant difference in the mean score of self-reported practice in three evaluation phases between two groups (*P* < 0.05). No significant relationship was observed between the difference in knowledge and self-reported practice scores and demographic factors.

**Conclusions:**

Both educational interventions regarding emergency management of TDIs-educational leaflet and oral presentation- were effective in increasing knowledge and self-reported practice of teachers in the short-term follow-up. In long-term evaluation, educational leaflet resulted in more positive changes in teachers’ knowledge compared to their self-reported practice.

**Electronic supplementary material:**

The online version of this article (10.1186/s12903-019-0823-4) contains supplementary material, which is available to authorized users.

## Background

Traumatic dental injuries (TDIs) are considered a public health problem due to their frequent occurrence in the society and negative impacts on the quality of life [1–3]. Facial trauma comprises 5% of all the traumatic injuries presented to hospitals. It is also prevalent among children and is an unpleasant experience for both parents and children [4].

The most common reasons for the occurrence of dentoalveolar injuries are falls, physical exercise and activities, blows from hard objects, and motor vehicle accidents [5, 6]. Falls are the most prevalent factor among all others, and due to an immature neuromuscular system in children, they are prone to these incidents [7]. The possibility of dentoalveolar injuries is high between the ages of 6 to 12 due to spending a great deal of time on different physical activities [8]. In 2003, Pacheco et al. reported that approximately 22–30% of children experienced dental trauma while they were attending school [9]. The higher prevalence of injuries leading to dental luxation in primary school children results from the immaturity and weakness of periodontal ligaments and elastic structure of the bones [10, 11].

Crown or root fracture, luxation injuries, avulsion, and injuries of the alveolar bone, soft tissue, gingiva and dental pulp are the most prevalent complications of TDIs [11–13]. These injuries are often cause considerable pain and discomfort as well as aesthetic, functional, psychological, and social problems and may ultimately affect the quality of life of the patients [14].

Many studies have found a significant deficiency in the teachers’ knowledge about TDIs [15–21]. For example, in a study in Brazil in 2016, the teachers claimed they acquired no specific knowledge about TDIs. The result of that study indicated that the Brazilian teachers’ knowledge and performance about acute permanent teeth’s injuries were based on imprecise information, and they mostly acted according to their wrong traditions and beliefs [16, 22]. If lay people, especially school staff, have sufficient knowledge and awareness of the first aid procedures and the necessity of emergency treatment of dental injuries, there will be a noticeable improvement in treatment outcomes since the best treatment results would be achieved when the injured teeth are treated immediately using appropriate procedures [22–24]. Parents, teachers, and other people who attend the scene at the time of an accident before the child is referred to the dentist have a key role in improving treatment outcomes and decreasing complications [24, 25].

Improvement of the knowledge of primary school teachers is extremely important to prevent and manage TDIs in schools and improve the prognosis of traumatized teeth. Few studies have showed improved elementary school teachers’ knowledge of emergency management of TDIs after intervention by some educational materials such as posters [10, 25–27] and lecture [17].

The present study was conducted to determine the effectiveness of an educational leaflet (a three-folded colorful sheet) in comparison with oral presentation (45-min lecture by a dentist with PowerPoint presentation) in promoting the primary school teachers’ knowledge and self-reported practice of emergency management of TDIs in Arak, Iran.

## Methods

### Study population and sampling

The present interventional study was conducted in primary school teachers. By census, all primary school teachers in Arak, Markazi Province, Iran (*n* = 664) were asked to participate in the study. Those who accepted to participate were assigned to two intervention groups of educational leaflet and oral presentation according to two geographic regions of the city.

### Baseline data collection

All primary teachers in both groups attended regular sessions according to their professional development program mandated by Department of Continuing Education, Ministry of Education. In the first stage, in coordination with the authorities of the Education Department of Markazi Province, teachers in both groups were asked to complete an anonymous self-administrated questionnaire in two different sessions.

### Interventions

**Fig. 1:**
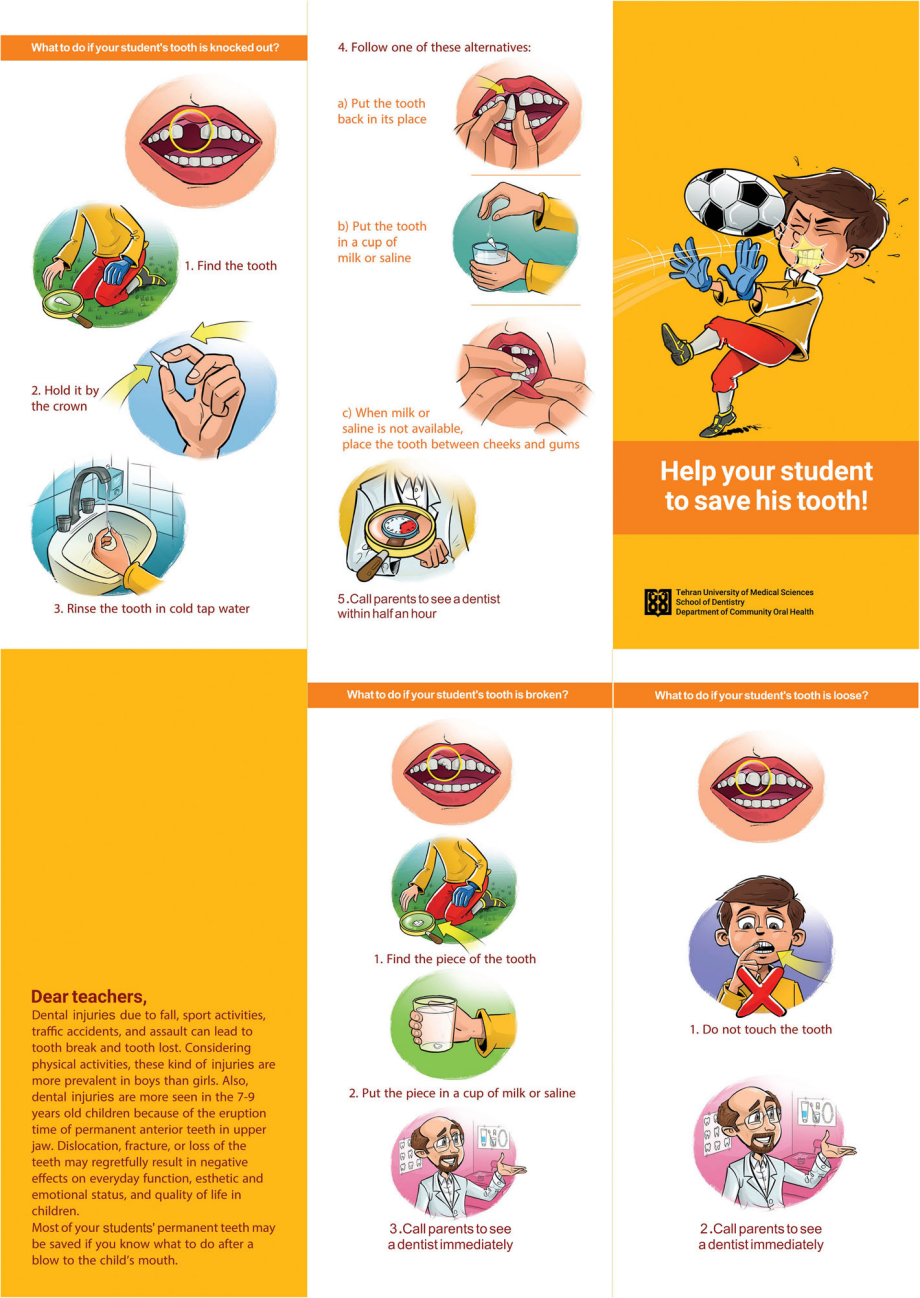
The educational leaflet provided as interventional tool regarding emergency management of TDIs

In the second stage, interventions, including an oral presentation and an educational leaflet whose contents were prepared based on the “Save Your Tooth” poster (IADT 2011) and the most recent scientific evidence, were applied [28, 29]. Both the oral presentation and the educational leaflet contained information about tooth fracture, luxation, and avulsion, and explanation about appropriate steps in the management of traumatized teeth. These materials were prepared in the native language (Farsi) and included colorful pictures (Fig. 1). In the oral presentation group (*n* = 341), a meeting was held and a brief explanation was provided about the purposes of the study; then, a 45-min oral presentation was delivered by one of the researchers. In the educational leaflet group (*n* = 323), a letter explaining the study was attached to the educational leaflet.

The teachers working in the city were categorized to the two groups by the Ministry of Education based on geographical location of the schools. We randomly assigned the interventions (oral presentation and educational leaflet) to these groups.

### Follow up data collection

In the third and fourth stages of the study, one and six months after the intervention, the same questionnaire was completed by the teachers. The answers were then scored and the data were statistically analyzed to compare the pre- and post-test results.

### Questionnaire

In addition to demographic data (age, employment status, education level, and work experience) and history of exposure to TDIs, the questionnaire (Additional file 1: Appendix 1) included the following items:

#### Knowledge

The teachers were asked to answer eight questions on the emergency management of TDIs using multiple-choice, “yes-no”, and “I do not know” answers. Two questions had two correct answers. Incorrect and “I do not know” answers scored = 0 and correct answers received a score of 1. By summing up the scores of eight questions, the knowledge score of each teacher was calculated (range: 0 to 10).

#### Self-reported practice

In this part, four paper cases (five questions) of TDIs were presented, each representing a case with a certain TDI. The cases were almost similar to the content of the oral presentation and the educational leaflet. Two questions had more than one correct answer: one question had three correct answers, and another one had two correct answers. Based on the teachers’ answers, the self-reported practice score (range: 0–8) was calculated as described for the knowledge score.

Teachers were requested to write a unique code on the top of their three questionnaires. This code was used to assess individual changes before and after the intervention.

In terms of face and content validity, a valid reference book [30], latest guidelines for the management of TDIs [28, 29], and similar previous studies [10, 17, 25–27] were used to collect the questions. Then, the questionnaire was piloted to assess its validity and reliability. Two experts in community oral health, one expert in pediatric dentistry, and one epidemiologist assessed the content validity of the questionnaire. The reliability of the questionnaire was evaluated and approved through test-retest on 15 primary school teachers from three schools of the city at an interval of 10 days. These teachers were excluded from the main study. The Kappa coefficient was above 70% for different questions.

### Ethics approval

Ethics approval was obtained from the Research Ethics Committee of Tehran University of Medical Sciences (code IR.TUMS.REC.1394.1383). This longitudinal and interventional study was completely voluntary and the responses were anonymous. Moreover, all respondents were free to leave the study in each phase. Informed consent was obtained from all participants in the beginning of the study. In the start page of the questionnaire, the participants were informed about the objectives and protocol of the study. Moreover, they were asked to present their informed consent by signing the bottom of this page.

### Statistical analysis

SPSS version 22 for Windows (SPSS Inc., Chicago, IL, USA) was used for statistical analysis. Repeated measures ANOVA was applied to analyze the data. In this test, knowledge and self-reported practice before and after the intervention were considered as repeated factors and demographic variables (age, employment status, education level, and work experience) and type of intervention were considered as between-subject factors. A linear regression model was used to analyze the relationship between independent variables and knowledge and self-reported practice scores. The association between knowledge and self-reported practice was explored using Pearson correlation coefficients. The level of significance was set at *p* < 0.05.

## Results

Of 664 teachers that were invited to participate in the study, 453 completed the baseline questionnaire. The number of participants who completed two follow-up questionnaires was 292 (*n* = 138 in oral presentation and *n* = 154 in educational leaflet group).

### Baseline results

The mean age of the participants in oral presentation and educational leaflet group was 36.83 ± 8.75, and 36.70 ± 8.64 years, respectively (Table 1). Most of the participants were officially employed by the Ministry of Education and had pre-university (college) education (Table 1). Moreover, 52.2% of the teachers in oral presentation group and 51.7% of the teachers in the educational leaflet group had a previous history of exposure to TDIs in their students.

**Table 1:** Background characteristics of teachers receiving education on TDIs management through educational leaflet (*n* = 138) and oral presentation (*n* = 154)

Background information		Educational leaflet group	Oral presentation group
		(*N* = 154)	(*N* = 138)
Employment status^a^ *No. (%)*	Official employment	79 (58.1%)	84 (56.0%)
	Contractual employment	7 (5.1%)	18 (12.0%)
	Adjunct employment	50 (36.8%)	48 (32.0%)
Educational level^a^ *No. (%)*	Diploma	25 (18.2%)	25 (16.6%)
	Pre-university	62 (45.3%)	61 (40.4%)
	Bachelor	40 (29.2%)	52 (34.4%)
	Master and higher degrees	10 (7.3%)	13 (8.6%)
Mean age *mean ± SD*		36/70 ± 8.64	36.83 ± 8.75
Mean work experience *mean ± SD*		13.60 ± 8.49	15.22 ± 9.08

^a^There were very few missing answers which were excluded from analysis

Before the intervention, the mean score of knowledge was 2.53 ± 1.77 and 2.78 ± 1.61in the educational leaflet and oral presentation group, respectively. At baseline, the mean score of knowledge had a significant relationship with the education level (*P* < 0.001). No other significant relationship was observed between the mean score of knowledge and other demographic variables.

Before the intervention, the mean score of self-reported practice was 3.67 ± 1.97 and 3.26 ± 2.12in educational leaflet and oral presentation group, respectively. There was no significant relationship between the mean score of self-reported practice and demographic variables.

### The findings of the interventions

#### Knowledge

Table 2 presents the mean score of knowledge in two groups in three different evaluation phases. In on-month follow-up, the mean score of knowledge in educational leaflet and presentation group increased to 5.85 ± 1.82 and 5.95 ± 1.66, respectively. Furthermore, the mean score of knowledge after 6 months was 4.49 ± 1.94 and 4.52 ± 1.84 in the educational leaflet and oral presentation group, respectively.

**Table 2:** Mean score of knowledge at different stages in teachers receiving education on TDIs management through educational leaflet (*n* = 154) and oral presentation (*n* = 138)

	Time of collecting the questionnaires	Mean + SD	*P*-value
Educational leaflet (*N* = 154)	Baseline	2.53 ± 1.77	0.84
	One-month follow-up	5.85 ± 1.82	
	Six-month follow-up	4.49 ± 1.94	
Oral presentation (*N* = 138)	Baseline	2.78 ± 1.61	
	One-month follow-up	5.95 ± 1.66	
	Six-month follow-up	4.52 ± 1.84	

In one-month follow-up, in both interventional groups, the mean score of knowledge was significantly higher compared to baseline and six-month scores (*P* < 0.001 and *P* < 0.001, respectively). Moreover, the mean score of knowledge was higher in six-month follow-up compared to baseline evaluation (*P* < 0.001) in both intervention groups. There was no statistically significant difference in the mean score of knowledge between the two groups in three evaluation phases (*P* < 0.05).

No significant relationship was observed between the difference in the knowledge score and demographic factors (age *P* = 0.55, work experience *P* = 0.68, employment status *p* = 0.31, educational level *p* = 0.78).

#### Self-reported practice

Table 3 shows the mean score of self-reported practice in two groups in three different evaluation phases. In 1 month after the interventions, the mean score of self-reported practice increased to 4.54 ± 1.63 and 4.19 ± 1.62 in educational leaflet and oral presentation group, respectively. Furthermore, after 6 months, the mean score of self-reported practice was 3.71 ± 1.51 and 3.60 ± 1.64 in educational leaflet and oral presentation group, respectively.

**Table 3:** Mean score of self-reported practice at different stages in teachers receiving education on TDIs management through educational leaflet (*n* = 154) and oral presentation (*n* = 138)

	Time of collecting the questionnaires	Mean + SD	*P*-value
Educational leaflet (*N* = 154)	Baseline	3.67 ± 1.97	0.27
	One-month follow-up	4.54 ± 1.63	
	Six-month follow-up	3.71 ± 1.51	
Oral presentation (*N* = 138)	Baseline	3.26 ± 2.12	
	One-month follow-up	4.19 ± 1.62	
	Six-month follow-up	3.60 ± 1.64	

In one-month follow-up, in both interventional groups, the mean score of self-reported practice was significantly higher compared to the baseline and six-month score (*P* < 0.001 and *P* < 0.001, respectively). Contrary to the mean score of knowledge, there was no significant difference in the mean score of self-reported practice between one-month and six-month follow-ups (*P* = 0.53). There was no statistical significant difference in the mean score of self-reported practice in three evaluation phase between the two groups (*P* < 0.05).

No significant relationship was observed between the difference in self-reported practice scores and demographic factors.

The Pearson correlation coefficient showed that self-reported practice had a weak correlation with knowledge in one-month and six-month follow-ups (*r* = 0.21, *r* = 0.33, respectively, *P* < 0.05).

## Discussion

The present study investigated the impact of two educational interventions, leaflet and presentation, regarding emergency management of TDIs on the knowledge and self-reported practice of primary school teachers in Arak, Iran. The results of short-term follow-up showed that both interventions were effective in improving the knowledge and self-reported practice of teachers in this regard. On long-term evaluation, educational leaflet resulted in more positive changes in teachers’ knowledge compared to self-reported practice.

The improved knowledge scores after one and six months in both groups may indicate the success of both interventions. Similar changes in the knowledge score were observed in the two groups, with the significantly highest score in the second (one-month) evaluation compared to the first (baseline) and the third (six-month) evaluation. Furthermore, the knowledge score was higher in the third follow-up compared to the baseline. The changes of the practice score indicated that this score improved in both groups after 1 month. In contrast to the knowledge score, the practice scores differed insignificantly between the baseline and the six-month evaluation in both group.

The grouping of the participants was according to the districts set by the Ministry of Education. Since the only basis for this grouping is geographic distribution, no significant background and demographic difference is expected between the two groups. The similarity of the knowledge and practice scores between the two groups at baseline supports this notion. The target group was considered as people who were mostly in close relationship with children subject to TDIs. Using a self-administered questionnaire resulting in self-reported data and a relatively short-term follow up can be considered the limitations of the present study.

The knowledge scores of the participants at bassline revealed some deficiencies in the teachers’ knowledge of TDIs. This finding is similar to that of some other studies reporting knowledge deficiency about dental trauma among teachers [11, 18, 31–34].

In the present study, four designed cases were used for assessment of the teachers’ practice regarding management of TDIs, which was deficient at baseline. This finding has also been noted in the similar studies conducted by Mohandas et al. in India [5], Letelier et al. in Chile [31] and Traebert et al. in Brazil [11]. Since practice efficacy is influenced by the level of knowledge, any deficiency in the teachers’ practice can be related to their insufficient level of knowledge.

The results of the present study showed that both educational methods of oral presentation and educational leaflet were capable of improving the knowledge and practice of teachers regarding TDIs management. Therefore, these two interventional methods can be taken into consideration for being utilized as educational tools in order to transfer useful information to the society.

It should be considered that the information presented in the form of educational leaflets should be attractive, compact, and comprehensive, and include the most basic points. Leaflets should be utilized for non-commercial purposes, and for conveying specific education to the audience who have no comprehensive information of the subject.

In the present study, the educational content of the oral presentation and the educational leaflet were similar, and both methods improved the teachers’ knowledge of TDIs as shown in one- and six-month follow-ups. The effectiveness of the educational leaflet could be attributed to its availability, enabling the teachers to have easy access to it. On the other hand, in oral presentation, both vision and auscultation are engaged in the learning process simultaneously that could be a possible reason for the effectiveness of this method.

The results indicated the effectiveness of both interventional methods in improving the teachers’ self-reported practice with no statistically significant difference between the two methods. In both groups, the highest practice score was acquired in the on-month follow-up. Hence, it can be concluded that part of the acquired knowledge might be forgotten over time, which could have an impact on people’s practice and result in deviation from the favorable level. According to a study by Young et al. in 2014, the effect of posters on the level of knowledge and practice of teachers is more prominent in a short time period of 2 weeks compared to long term [25]. Furthermore, it has been shown that the effect of one single oral presentation on TDIs management is insignificant in lay people in the long term [35].

In the present study, although the level of knowledge in both groups increased after the intervention, it was still below an acceptable level. In addition, a single educational session cannot produce an acceptable level of experience in teachers to manage TDIs in the long term [26]. In a study by De Lourdes Vieira Frujeri et al., although an educational intervention was performed, a high percentage of subjects claimed that they were not able to place a tooth that was taken out by avulsion [26]. Thus, it seems that continuous educational sessions are required in this regard.

A study limitation was that almost one third of the enrolled participants were lost to follow-up. The main reasons for dropouts were the voluntary nature of participation in the study, lack of rewards to respondents, and the participants’ lack of time, which were other than the content of the questionnaire, outcomes of the study, and interventions; moreover, the dropout rates were similar in both study groups. Additionally, the baseline data showed no difference between the drop-outs and those who participated in the final follow-up. However, caution should be exercised in interpreting the results.

## Conclusion

Both educational interventions regarding emergency management of TDIs (educational leaflet and oral presentation) were effective in improving knowledge and self-reported practice of teachers in the short-term follow-up. On long-term evaluation, the educational leaflet resulted in more positive changes in teachers’ knowledge compared their self-reported practice.

## Additional file

Additional file 1: Appendix 1: The questionnaire used as collecting data tool regarding emergency management of TDIs. (DOCX 21 kb)

## Data Availability

Not applicable.

## Abbreviations

SPSS: Statistical Package for the Social Science
TDIs: Traumatic Dental Injuries
