# Supplementary material for: Effect of two educational interventions on primary school teachers’ knowledge and self-reported practice regarding emergency management of traumatic dental injuries
Source: BMC Oral Health. 2019 Jun 27;19:130. doi: 10.1186/s12903-019-0823-4 (PMC6598348; doi:10.1186/s12903-019-0823-4)
Supplement: Supplementary file 1 — Appendix 1: The questionnaire used as collecting data tool regarding emergency management of TDIs. (DOCX 21 kb) [file 12903_2019_823_MOESM1_ESM.docx]

Appendix

Dear teachers,

The goal of this study is to evaluate your awareness on dental injuries management in 6-12-year-old students.

Your genuine responses and cooperation are greatly appreciated. This questionnaire will be given to you three times. You are free to take part in this study. Your responses are anonymous. However, for comparison of your responses, we request that you provide some demographic information.

Please write a specific code and remember it (last four numbers of your cell phone).

Code ….

Date ….

Thank you for your genuine responses and cooperation.

Signature

Age ……

Year number of work experience ……

Employment status

Official Contractual Others

Education level

High school diploma College degree University degree and higher

Have you ever encountered a dental trauma in a child?

Yes No

Have you already participated in any educational course on dental trauma management?

Yes No

Do you think you have enough information in this area?

Yes No

Would you like to learn more about dental injuries management??

Yes No

**Knowledge Questions:**

If tooth injuries are preventable?

Yes No I do not know

If the tooth is broken, can the broken part be attachable again?

Yes No I do not know

Can a knocked out primary tooth be replaced?

Yes No I do not know

Can a knocked out permanent tooth be replaced?

Yes No I do not know

Where is the first place you would take the child after dental trauma?

Medical office Dental office Hospitals’ emergency room I do not know

What is the best time for replacing a knocked out tooth?

Immediately Less than half an hour Within few hours I do not know

Which is the best way to clean the knocked out tooth before replacing it? Using ....

Toothbrush Tap water Saline Milk The tooth must not be cleaned

I do not know

How would you carry the knocked out tooth to dentist? In ....

Ice Tap water Milk Child’s mouth Handkerchief I do not know

**Self-reported practices:**

**In the following part, there are four situations which can lead to dental trauma. After reading each situation precisely, please choose the correct answer.**

Case1-A 9-year-old girl falls on the ground while playing and her upper incisor is fractured. There are no other injuries. What is the first thing that you will do?

I will do nothing, if there is no pain or bleeding.

I will find the fragment and immediately take the child to dentist.

I will find the fragment and immediately call parents to see a dentist.

I do not know.

Case 2-A 12-year-old boy fall on the ground while playing soccer. His mouth is covered with blood and his upper incisor does not present at its place. He has no other injuries. What is the best action that you will take?

I will give him a handkerchief to press on it for stopping bleeding.

I will find the tooth immediately, wash the tooth, replace it, and call parents to see a dentist.

I will find the tooth immediately, store it in a fluid, and call parents to see a dentist.

I will find the tooth, give it to the child to store it in his mouth, and call parents to see a dentist immediately.

I do not know.

Case 3- A 10-year-old child fall on the ground while playing and lose his consciousness. What is the first action that you take in this situation?

I will try to awaken him.

I will call the medical emergency service immediately.

I do not know.

Case 4- A 10-year-old child bump another child while playing and when you look in to his mouth you find that one of his incisors is displaced to intraoral. He has no other injuries. What will you do in this situation?

I would try to replace the tooth and call parents to see a dentist immediately.

I would not touch the tooth and call parents to see a dentist immediately.

I would not do anything, just call parents to see a dentist immediately.

I do not know.
